# A New Approach for Assessing Sleep Duration and Postures from Ambulatory Accelerometry

**DOI:** 10.1371/journal.pone.0048089

**Published:** 2012-10-24

**Authors:** Cornelia Wrzus, Andreas M. Brandmaier, Timo von Oertzen, Viktor Müller, Gert G. Wagner, Michaela Riediger

**Affiliations:** 1 Research Group Affect Across the Lifespan, Max Planck Institute for Human Development, Berlin, Germany; 2 Center for Lifespan Psychology, Max Planck Institute for Human Development, Berlin, Germany; 3 Psychology Department, University of Virginia, Charlottesville, Virginia, United States of America; 4 Max Planck Institute for Human Development and German Institute for Economic Research, Berlin, Germany; University of Adelaide, Australia

## Abstract

Interest in the effects of sleeping behavior on health and performance is continuously increasing–both in research and with the general public. Ecologically valid investigations of this research topic necessitate the measurement of sleep within people’s natural living contexts. We present evidence that a new approach for ambulatory accelerometry data offers a convenient, reliable, and valid measurement of both people’s sleeping duration and quality in their natural environment. Ninety-two participants (14–83 years) wore acceleration sensors on the sternum and right thigh while spending the night in their natural environment and following their normal routine. Physical activity, body posture, and change in body posture during the night were classified using a newly developed classification algorithm based on angular changes of body axes. The duration of supine posture and objective indicators of sleep quality showed convergent validity with self-reports of sleep duration and quality as well as external validity regarding expected age differences. The algorithms for classifying sleep postures and posture changes very reliably distinguished postures with 99.7% accuracy. We conclude that the new algorithm based on body posture classification using ambulatory accelerometry data offers a feasible and ecologically valid approach to monitor sleeping behavior in sizable and heterogeneous samples at home.

## Introduction

Self-reported sleep duration has been closely linked to diverse illnesses and mortality risk [1]–[3]. Interest in studying sleep and sleep disturbances within the general population is therefore thriving. Self-reported sleeping behavior can be obtained from large population samples, yet relies on subjective information. Objective assessments in sleep laboratories offer many advantages, but are often carried out with small samples; and their comparability to daily life, that is, their ecological validity, is disputed [4], [5]. Thus, representative samples and ecologically valid measurements require approaches to monitor sleeping behavior outside the laboratory. So far, the preferential approach is actigraphy–that is, determining sleeping and waking behavior from physical activity mainly measured through wrist movements while participants pursue their normal daily routines in their home environment [4], [6]. Here, we demonstrate that ambulatory assessment of whole-body physical activity and body postures through accelerometry offers another approach to measure people’s sleeping behavior in terms of both duration and quality in an objective, unobtrusive, reliable, and valid way.

### Assessment of Sleep Behavior Using Self-Reports, Polysomnography, or Wrist Actigraphy

Sleep duration and sleep quality have been assessed with self-reports [7], [8], polysomnography, that is, psycho-physiological measurements of sleep in sleep laboratories [9], or actigraphy [4], [6], [10]. Self-reports of sleep duration and quality depend on people’s ability and compliance to accurately remember and retrospectively report their sleeping behavior [4]. Objective assessments of sleeping behavior via polysomnography may also diverge from the participants’ typical sleep because of the unusual sleeping environment and the obtrusive measurement equipment attached to the sleeper, which both might affect the sleeping behavior [4], [5], [11]. Ideally, sleeping behavior should thus be measured objectively and unobtrusively, yet within the participants’ natural environment, for example with actigraphy. Sleep-focused actigraphy mainly determines people’s sleeping and waking times based on wrist movements, which are less pronounced when people are asleep [4], [6], [11], [12]. Actigraphy “has reasonable validity and reliability in assessing sleep–wake patterns in normal individuals with average or good sleep quality” [6]. Yet, actigraphy based on wrist movements is less well suited for samples other than the common samples focusing on young, healthy adults and for determining specific indicators of sleep quality and sleeping posture due to being based on wrist movements only. In addition, we acknowledge that, in contrast to actigraphy, the combined polysomnographic assessment of electroencephalography (EEG; i.e., brain activity), electroocculography (EOG; i.e., eye movements), electromyography (EMG; i.e., muscle movements), and airflow of breathing allows the precise measurement of sleeping stages (e.g., REM phases or slow wave sleep).

We propose that many important indicators of sleep duration and quality usually obtained from polysomnography can also be assessed with algorithms developed for whole-body physical activity measurements. These indicators include: (a) time in bed; (b) total sleep time; (c) sleep efficiency, that is, percentage of time in bed spent asleep; (d) sleep latency, that is, time in bed until asleep; (e) number of awakenings; (f) sleeping postures; (g) number of posture changes; (g) number of postures that last longer than 15 mins; and (i) average activity during sleep [9], [11].

Time in bed and time asleep refer to aspects of sleep duration that can differ greatly between individuals [9], [13]. Sleep efficiency and sleep latency are indicators of sleep quality that are computed from examining time in bed and time asleep. Greater sleep efficiency is characterized by less time awake during the night and shorter sleep latency by a shorter time span between going to bed and falling asleep. Both measures are indicative of healthy sleeping patterns. Similarly, fewer awakenings during the night indicate higher sleep quality, although even healthy people wake up on average four to six times per night [14]. Normal sleeping patterns thus involve a certain amount of physical activity during the night. This is reflected, for example, by healthy people changing their posture, for instance, from lying on the back to lying on one side, between 10 and 30 times per night [15]. Nonetheless, too many posture changes occurring shortly after each other, that is, tossing around or staying in the same posture for less than 15 minutes at a time, indicate restless sleep of worse quality [14]–[16].

We propose that multiaxial acceleration sensors attached to the torso are well suited for the ambulatory assessment of the aforementioned indicators of sleep duration and quality because they allow for determining the orientation of the body and are less sensitive to limb movements. The purpose of our research was to demonstrate the validity of this new approach to monitor sleep in natural environments in two respects. First, indicators of sleep duration and quality derived from ambulatory accelerometry should converge with participants’ self-reported sleep duration and quality (convergent validity). Second, sleep indices derived from ambulatory accelerometry should replicate well-documented differences in sleep duration and quality (external validity). We did not compare participants’ sleeping behavior to polysomnographic measurements because the comparability between polysomnographic assessments in the sleep laboratory and less cumbersome accelerometry assessments in participants’ homes, that is, familiar environment, has been questioned [4], [5], [11].

### Age-Related Differences in Sleep Duration and Quality

Individual differences related to age are among the best-replicated patterns of differences in sleeping behavior. Previous representative studies from the UK and the USA, for example, report an average self-reported sleep *duration* of about 7 hrs and describe a decrease of sleep duration from young adulthood to middle adulthood followed by a leveling off or a slight increase in sleep duration after retirement [7], [13]. This converges with meta-analysis findings of age differences observed in laboratory studies [9].

Findings are somewhat less consistent when it comes to age-related differences in sleep *quality*. Generally, compared to younger people, older people are less active during sleep, as indicated by fewer posture changes and less activity in general [14], [17]. At the same time, most laboratory and survey studies report an age-related decrease of sleep quality as indicated by lower sleep efficiency, or more time awake while in bed, and a higher frequency of sleep disturbances [8], [9], [18]. Recently, however, Vitiello [19] reviewed that lower average sleep quality in old age may be primarily due to age-related declines in the general health status, and that healthy older people are not more prone to sleep disturbances than younger people.

In the current study, we test whether accelerometry of body movements during the night offers feasible, relatively unobtrusive, reliable, and valid measurements of people’s sleeping duration and quality in their natural environment. We extend previous methodological work on actigraphy during sleep twofold: (1) by focusing on an age-heterogeneous sample ranging from adolescence to old age because the applicability of actigraphy in samples heterogeneous in age and sleep quality still needs to be shown [4], [6] and (2) by proposing a new methodological approach to determine sleep postures and indicators of sleep quality based on changes in sleep postures. We validate our approach in two ways. First, we predict convergent validity between accelerometry-based indicators and subjective ratings both of sleep duration and quality. Second, we test external validity by replicating age differences in sleep duration and quality. We hypothesize that (a) accelerometry-based sleep duration is longer for younger and older adults than for middle-aged adults; (b) general activity is lower with higher age; and (c) accelerometry-based sleep quality tends to be lower the older the participants are, however, these age differences are assumed to be attributable to age differences in health.

## Materials and Methods

### Participants

Ambulatory accelerometry was obtained from a German sample of 92 people recruited in Berlin in 2008 by a fieldwork agency. Participants were on average 42.4 years old (*SD* = 19.0; range 14.7–83.2 years), and 26.1% held a college or university degree. Participants were approximately stratified with respect to gender (45% men) and across seven age groups (14–18 years: *n* = 10; 19–29 years: *n* = 18; 30–39 years: *n* = 16; 40–49 years: *n* = 14; 50–59 years: *n* = 12; 60–70 years: *n* = 15; 70–83 years: *n* = 7). Accelerometry data from 3 female and 9 male participants were excluded due to measurement problems (e.g., sensor cables were disconnected, sensor position shifted). Participants belonged to the following age groups: adolescents and young adults n = 4, middle-aged adults n = 5, older adults n = 3. They did not differ significantly from the remaining participants in age, *χ^2^* = 0.95, df = 2, *p* = .62, and only marginally with respect to gender, *χ^2^* = 3.85, df = 1, *p* = .05. In contrast to previous studies that used participants’ data if at least 4 hrs of recording were available [20], our exclusion criteria were comparatively strict because most of the indicators of sleep duration and sleep quality in the current study referred to the whole night, for example, sleep duration, sleep efficiency, or the number of times people arose at night. Compared to previous studies, we obtained similarly reliable assessments because all participants provided at least 4 hrs of continuous recording.

### Procedure, Ethics Statement, and Measurements

#### Measurement of activity and postures using accelerometry

**Figure 1:**
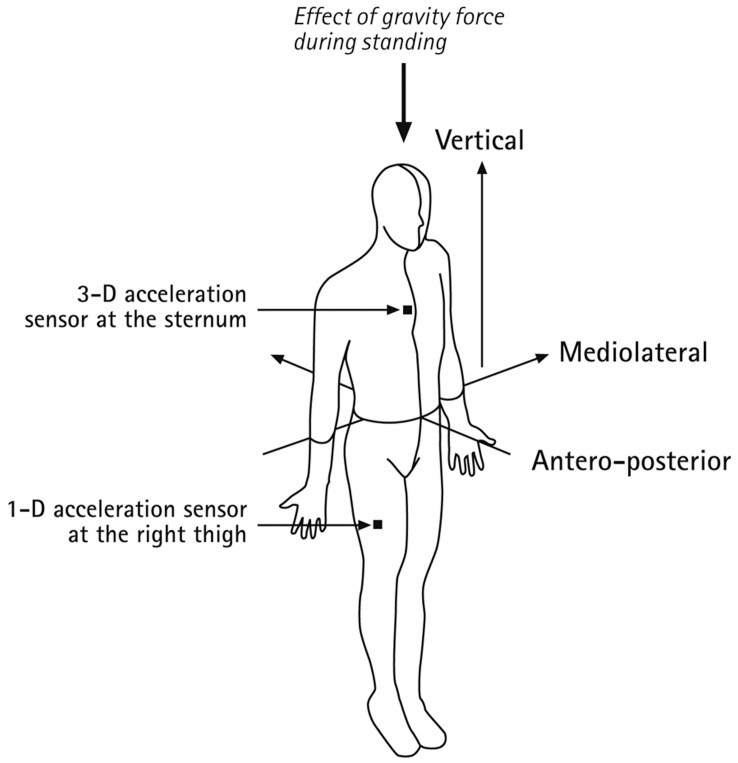
Labeling of body axes and placement of acceleration sensors.

Participants came to the laboratory to receive the technical devices and instructions on their use. After explaining the purpose, assessment devices, and procedure of the study, written informed consent was obtained from the participant and the parents of underage participants, and a portable biosignal recorder (Varioport from *Becker Meditec* company) with acceleration sensors were attached to them. Trained experimenters placed a three-dimensional (3-D) acceleration sensor on the sternum and a one-dimensional (1-D) acceleration sensor on the right thigh, parallel to the antero-posterior axis (see Figure 1). The analysis of the accelerometer data was based on four recorded channels corresponding to the axes of the two sensors. The 24-hr ambulatory monitoring also included measurements of heart rate (ECG) and breathing frequency. In the interest of brevity, we restrict the description of methods to those details that are relevant for the purposes of this article, the validation of a parsimonious measurement of sleep indices.

Participants completed a standardized sequence of motions and postures for the development of the classifying algorithms. A decision tree was used to classify physical activity into distinct postures, that is, to decide which posture is likely to occur given the observed pattern of physical activity [21], [22]. The laboratory procedure for developing the decision tree included 40 s walking at normal pace, 50 s walking up stairs, 40 s walking down stairs, 40 s walking at fast pace, 40 s standing, 40 s sitting, 40 s lying on the right side, 40 s lying on the left side, and 180 s lying on the back. After answering a few questionnaires and completing a brief experiment irrelevant for the purposes of this paper, participants were released to continue their daily life for an average of 25.8 hrs (*SD* = 0.8 hrs; *min = *22.3 hrs, *max = *29.8 hrs). During this ambulatory-assessment phase, acceleration data were continuously recorded at a sampling rate of 64 Hz. Here, we focus on the measurements obtained during the evening and nighttime. Participants returned to the laboratory the next day with the bio-monitoring system and had to answer a few final questionnaires that included, among other things, items regarding their usual sleeping behavior, their sleep during the previous night, and their general health, as reported below. They were reimbursed with 150 € (approximately $180). The Ethics Committee of the Max Planck Institute for Human Development approved the study.

#### Development of the decision tree and classification of acceleration data into indices of sleep duration and quality

A classification algorithm of body posture and posture changes was developed to derive indices of sleep duration and quality from the accelerometry data recorded between 8 p.m. and 10 a.m. Next, the principles of the classification are explained before the computation of indices for sleep duration and quality is explicated.

Recorded acceleration data contained both deflections of the acceleration sensors due to the gravitational force and the active movements of participants [23]. The gravitational force affects acceleration sensors attached to the body differently depending on the position of the body relative to the earth’s surface. For example, gravitational force affects the body along the vertical axis during standing and along the antero-posterior axis when lying on the back (Figure 1).

The decision tree made binary decisions on body posture that were hierarchically ordered. First, body posture was classified as lying if the upper body deviated more than 40° from the upright position, that is, if the gravitational force exerted less than −0.66 g on the vertical axis (Figure 1). Otherwise, information on how gravitational force affected the 1-D acceleration sensor attached to the thigh was used to distinguish between sitting (>0.5 g) and standing (<0.5 g). These thresholds and the prediction accuracy of the decision tree were derived by 10-fold cross-validation on the laboratory data, where postures were known during the standardized sequence of motions and postures. The discrimination between standing, sitting, and the different supine postures was accurate in 99.69% of all cases over all participants–constituting high reliability.

To determine the body posture during the night, effects of the gravitational force were again separated from the active movements of participants by computing the median and standard deviation of acceleration data in sliding windows of 5 s in length. Median values were then used to classify postures using the thresholds from the decision tree developed from the standardized postures observed in the laboratory. The decision tree distinguished between segments when participants were standing/walking, sitting, or lying supine, that is, horizontal. Body posture of participants was classified continuously in 5 s windows across the entire nighttime (8 p.m. to 10 a.m.).

As in previous studies, most indices on sleep duration and quality were only calculated for the time when participants were supine. As indicators of sleep duration, we computed the total time supine and the total sleep time. We calculated sleep efficiency, sleep latency, average activity, and the number of posture changes per hour (including getting up) as indicators of sleep quality. Tables 1 and 2 provide detailed descriptions of the computation of all indices on sleep duration and quality derived from acceleration data.

**Table 1:** Indicators of sleep duration derived from accelerometry.

	Accelerometry indicator	Computation	Unit
Time in bed	Time supine	time between beginning and end of supine posture for at least 50 s(ten 5 s windows) minus time sitting or standing	Hour
Total sleep time	Time asleep derived from accelerometry	time supine with less activity than the average minimumwhile lying supine in the laboratory (0.15 g^a^)	Hr

**Table 2:** Indicators of sleep quality derived from accelerometry.

	Accelerometry indicator	Computation	Unit
*Sleep quality – activity focused*			
Average activity	Average activity	mean square root of the sum squared values of four channels of acceleration sensors	*g* ^a^
Sleep latency	Still position latency	duration from onset of *time supine* until *time asleep derived from accelerometry* forat least 3 consecutive minutes	Minutes
*Sleep quality – posture focused*			
Sleep efficiency	Sleep efficiency based on turning	percentage of time without turning and rises relative to overall *time supine*	Percentage
Awakenings	Rises	postures with a threshold value of less than 40° gradient relative toupright position (> -.66 g on vertical axis)	Count
Sleeping posture	Posture	orientation of the thorax when body is in supine posture: deviation fromupright position >40°	Categories
		lying ventral: −45° to +45° around vertical axis	
		lying right: +45° to 135° around vertical axis	
		lying dorsal: −135° to +135° around vertical axis	
		lying left: −45° to −135° around vertical axis	
Posture changes	Posture changes	detected by continuously classifying the upper body rotation around the vertical axiswith a sliding circular mean across the last 5 s segment; change was classified if theabsolute difference from the currently observed angle (e.g., 0°, which means lying flaton the stomach) and the sliding circular mean angle differed by more than 30°.	Count
		This angle-based adaptive algorithm captures the sleeping posture better than a fixed threshold that discerns between sleeping postures; using fixed thresholds to determine sleeping posture often lead to a fast jitter between sleeping postures when close to the threshold because of intraindividual variations of sleeping behavior and varying sensor position; jitter suggests changes in sleeping postures, although only minor body shifts occurred, and leads to an overestimation of posture changes. The adaptive algorithm compensates for this by allowing for angular variations within sleeping postures anddetects a change of sleeping posture only if an angular change of 30° or more wasfound between two segments of analysis.	
	Duration in postures	mean duration of staying within specific posture	Minutes
Postures longer than 15 mins	Postures longer than 15 mins	Count of postures with duration longer than 15 minutes	Count

*Note*. ^a^standard gravity unit 9.81 m/s^2^.

To determine sleeping postures during supine phases, for example, on the right side or ventral, a newly developed angle-based segmentation algorithm (see Table 2) was developed. The angular orientation of the upper body along the vertical axis, that is, the sleeping posture, was constantly tracked. The algorithm continuously calculated a sliding circular mean. Whenever the current orientation estimate differed more than 30° from the sliding mean of the angle, a segment boundary was set that indicated that the sleeping posture had changed. The estimation of the sleeping posture changes with fixed thresholds for sleeping postures would be prone to detect a change in posture even if it hardly changed. This occurs when sleepers engage in sleeping postures near fixed thresholds. In contrast, the segmentation with flexible, angle-based thresholds allowed the algorithm to detect posture changes in a robust way. The body orientation within a segment, that is, the sleeping posture, was derived from the circular mean orientation across the segment. Lying ventral, that is, on the stomach, was defined to express an average rotation of 0° on the vertical axis. An orientation of −45° to +45° was classified as lying ventral, +45° to 135° as lying on the right side, −135° to +135° as lying dorsal, and −45° to −135° as lying on the left side.

#### Self-reported sleep duration, sleep quality, and health

Participants answered the questions “How many hours do you usually sleep during weekday nights/… during weekend nights?” These questions are identical to the questions used in the 2008 assessment of the large German Socio-Economic Panel Study (SOEP) [24]. We focus on self-reported sleep duration on weekday nights because the current study took place only on weekday nights. Participants indicated how well they usually sleep by using a scale ranging from 1 “poor” to 5 “very good.” They also reported how many hours they slept during the previous night (i.e., during the night of study participation). They rated whether the sleep duration of the previous night was sufficient compared to usual nights (1 “too short” to 5 “absolutely sufficient”) and whether their sleep had been restful (0 “not at all” to 4 “absolutely”). In addition, participants reported whether they had changed their daily routine during the 24-hr monitoring phase by using open response formats.

Finally, participants rated their health status on a 7-point scale ranging from 1 “not at all healthy” to 7 “absolutely healthy” and reported how often they had visited a doctor during the previous 12 months using the categories 0 “never,” 1 “once,” 2 “2–5 times,” 3 “5–10 times,” and 4 “more than 10 times.”

## Results

Based on participants’ open-answer reports on whether they had changed their daily routine during study participation, the subjective feasibility of the sleep monitoring was very high. Overall, only five persons (5.3%) reported that they felt slightly restricted during the night because they felt uncomfortable by not sleeping in a ventral posture due to study equipment. Nonparametric Mann-Whitney *U*-test comparisons to the remaining participants revealed that there were no significant differences in self-reported sleep duration, *M* = 6.90 hrs, *SD* = 0.89, Z = −0.65, *p* = .52; total sleep time derived from accelerometry, *M* = 6.77 hrs, *SD* = 2.06, Z = −1.10, *p* = .28; ratings of sleep as sufficient, *M* = 4.40, *SD* = 1.34, Z = −1.10, *p* = .27; or as restful sleep, *M* = 2.40, *SD* = 1.51, Z = −0.05, *p = *.96. Importantly, these five participants lay, on average, 35.4% of the night duration in a ventral posture, that is, on their stomach, despite their self-reported interference with lying in a ventral posture.

We next present evidence regarding convergent validity between the indicators of sleep duration and sleep quality derived from ambulatory accelerometry and from self-reports. Following this, we describe analyses regarding the external validity of the ambulatory accelerometry indices for sleeping behavior, denoted by patterns of age-related differences of indices.

### Convergent Validity Regarding Sleep Duration

Table 3 presents correlations between self-reported usual sleep duration (*M* = 6.95 hrs, *SD* = 0.95), self-reported sleep duration on the study night (*M* = 7.29 hrs, *SD* = 1.39), time supine derived from accelerometry (*M* = 8.12 hrs, *SD* = 1.25), and total sleep time derived from accelerometry (*M* = 7.51 hrs, *SD* = 1.33). Agreement between self-reported usual sleep duration and self-reported sleep duration on the study night was moderate (Table 3). Agreement between self-reported sleep duration on the study night and indicators derived from acceleration sensors was even higher. That is, people who reported more hours of sleep on the study night were also observed to be asleep longer based on accelerometry data, that is, in a supine posture with little activity, *r* = .50, *p*<.001. When data from one person who reported to have slept less than 3 hrs was excluded, the agreement between reported and observed sleep duration increased significantly, *r* = .68, *p*<.001, *Z* = 2.57, *p* = .01. Importantly, the associations between the various measures of sleep duration reported in Table 3 were not significantly moderated by participants’ age (all age interaction terms *p*>.10 in regression analyses also including the age main effect; no significant model improvements, *p*>.20). The results indicated that the time classified as sleeping based on the supine body posture and little activity was similar to sleep duration reported for that night and only slightly exceeded self-reported usual sleep duration: The total sleep time based on accelerometry did not differ significantly from the self-reported sleep duration during the study night, *t*(79) = 1.16, *p* = .25. Compared to usual sleep duration, participants slept somewhat longer during the night of the study: 20.4 mins more based on the self-report, *t*(77) = −2.56, *p* = .012; 33.6 mins more based on accelerometry, *t*(77) = −3.37, *p* = .001.

**Table 3:** Association between self-reported sleep duration and time supine during study night.

	Self-reported general sleep duration	Self-reported sleepduration on study night	Time supine derived from accelerometry
Self-reported sleep duration on study night	.47		
Time supine derived from accelerometry	.32	.58	
Total sleep time derived from accelerometry	.27	.50	.74

*Note*. All results *p*<.01; Associations were not significantly moderated by participants’ age, *p*>.10.

### Convergent Validity of Sleep Quality

Participants rated their usual sleep quality as rather high, *M* = 3.69, *SD* = 0.91 (“5” indicating “very good”) and judged their sleep on the study night as rather sufficient, *M* = 3.99, *SD* = 1.10 (“5” indicating “absolutely sufficient”), and restful, *M* = 2.39, *SD* = 1.12 (“4” indicating “absolutely restful”). The associations between self-reported sleep quality and indicators of sleep quality derived from accelerometry provided indication for convergent validity (Table 4): People, who were longer supine or asleep according to the accelerometry, rated their previous night’s sleep as more sufficient. Also, people who were less active during the night rated their sleep as more sufficient and more restful. There were no significant associations between still-position latency and self-reported sleep quality that night.

**Table 4:** Associations between objective sleep indicators and self-reported sleep quality as well as age.

		*M* (*SD*)	General sleep quality	Sufficient sleep on study night	Restful sleep onstudy night	Age
*Activity related indicators*						
Time supine	8.12 (1.25)		.06	.30**	.06	β_age_ = −.07
						β_age^2^_ = .27*
Total sleep time derived	7.51 (1.33)		.12	.18*	.06	β_age_ = .02
from accelerometry (h)						β_age^2^_ = .31**
Average activity	0.004 (0.001)		−.00	−.25^**^	−.19*	β_age_ = −.21*
Still position latency (mins)	4.22 (5.07)		.04	−.14	−.01	β_age_ = −.12
*Posture related indicators*						
Sleep efficiency (based on turning)		0.70 (0.20)	.19*	.12	−.02	β_age_ = −.17†
Rises in first hour		0.35 (0.60)	.17	−.01	−.04	β_age_ = −.18*
Rises per hour		0.09 (0.14)	.16	−.06	−.06	β_age_ = −.11
Posture changes per hour		3.05 (1.09)	.22*	−.06	−.02	β_age_ = −.19*
Average duration in posture (mins)		22.82 (11.50)	−.24*	.05	.04	β_age_ = .15
Number of postures per hour kept longer than 15 mins		1.13 (0.30)	.24*	.09	.19*	β_age_ = −.18*

*Note*. ** *p*<.01,

* *p*<.05,

† *p* = .06.

Overall, Table 4 shows that posture-related indicators of sleep quality were more strongly related to self-reported *general* sleep quality than to self-reported sleep quality *during the study night*: Greater sleep efficiency as indicated by accelerometry, that is, a higher percentage of time in bed with little activity based on turns, was associated with better general self-reported sleep quality. Seemingly contrary to our assumptions, people who remained a shorter time in a specific posture or who had relatively more posture changes per hour also reported a higher general sleep quality. However, follow-up analyses showed that the number of posture changes also revealed a quadratic association with self-reported general sleep quality, β_linear_ = .26, *p* = .02, β_quadratic_ = −.21, *p* = .05, *F*(2, 76) = 3.84, *p* = .03. Both participants with relatively few or relatively many posture changes per hour reported less general sleep quality compared to participants with an average number of posture changes. That is, although some activity and posture change is normal, relatively turbulent sleep relates to a reported worse sleep quality. Therefore, people who had more phases where they remained in a specific posture for longer than 15 mins rated higher on both general sleep quality and restfulness on the study night. Importantly, the associations between self-reported sleep quality and indicators of sleep quality based on physical activity were also not significantly moderated by participants’ age (all age interaction terms, *p*s >.10, in regression analyses that also included the age main effect; no significant model improvements, *p*s >.20).

The prevalence of sleeping postures derived from the accelerometry data was similar to those observed in laboratory studies [25], [26]. Most people–62.5% of the participants–first lay on their back, 21.2% first lay on the right side, 13.8% first lay ventral, and 2.5% first lay on their left side. Later on, the dominant posture changed: On average people spent 45.1% of the time supine on their left side, 28.6% of the time on their stomach, 13.4% of the time on their back, and 11.6% of the time on their right side. Both the first posture in bed and the percentage of supine time spent in specific postures were not significantly related to the subjective ratings of sleep quality (all *p*s >.20).

In sum, the time of supine and average activity based on accelerometry during the study night was related to participants’ rating that specific night as sufficiently long and restful the next day. The number of posture changes was not significantly related to how they judged their sleep the night before, but it was related to general judgments of sleep quality.

### External Validity: Age Differences in Sleep Duration and Quality

We compared sleep duration determined by accelerometry to self-reported sleep duration assessed in the nationally representative sample of 19,618 participants (*M*_age_ = 49.5, SD = 17.7; range 18–99 years; 48% male) in the 2008 assessment of the German SOEP [24]. The SOEP study is comparable to the British Household Panel Study [27]. The SOEP study assessed self-reported usual sleep duration for the first time in 2008 and thus provides comparative data on sleep duration in Germany. Previously, such data were only available for the UK and USA [7], [13].

**Figure 2:**
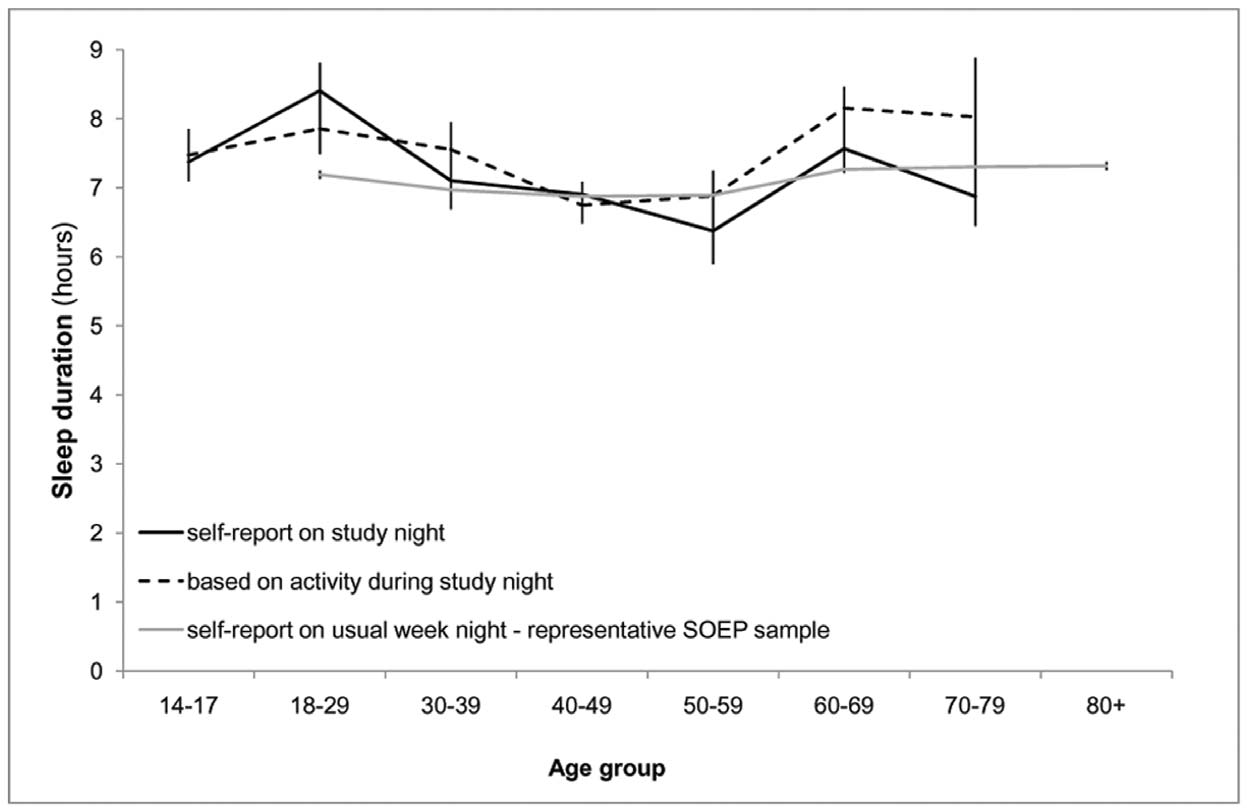
Age differences in sleep duration. Lines represent age differences in self-reported usual sleep duration in the representative SOEP survey for 2008, in self-reported sleep duration during the study night, and in sleep duration based on activity measures during the study night.

Consistent with our hypotheses, we observed a decreased sleep duration in middle adulthood compared both to adolescence and old adulthood; in self-reported duration on the study night, *F*(2,77) = 3.79, *p* = .027, β_age_ = −.25, *p* = .026, β_age^2^_ = .20, *p* = .071; in sleep duration obtained from acceleration-sensor data on the study night, *F*(2,77) = 4.09, *p* = .02, β_age_ = .02, *p* = .83, β_age^2^_ = .31, *p*<.01; as well as in the representative SOEP sample, *F*(2,19615) = 180.21, *p*<.01, β_age_ = .04, *p*<.01, β_age^2^_ = .12, *p*<.01 (Figure 2).

Age differences in sleep quality also revealed the expected pattern (Table 4). Older participants were less active during sleep (i.e., moved less), got up less often during the first hour of sleep, and showed less frequent posture changes, but they also tended to show a decrease in sleeping efficiency, that is, the sleep time relative to the time in bed. Since decreased sleep efficiency is assumed to be less a matter of age, but rather of increasing prevalence of illnesses, we conducted follow-up analyses regarding the participants’ health status. They confirmed the expected associations between higher sleep efficiency and fewer number of medical consultations during the last year, *r = *−.22, *p* = .03, or higher self-reported health status, *r = *.20, *p* = .04. At the same time, higher age was associated with more frequent medical consultations, *r = *.28, *p*<.01, and lower self-reported health status, *r = *−.41, *p*<.01. When controlling for the participants’ health status, there was no significant association between age and sleep efficiency (*p*>.45). Therefore, not higher age per se, but rather health problems were related to less sleep efficiency.

In sum, accelerometry-based indicators of sleep duration and quality confirmed and refined knowledge on age-related differences in sleep duration and quality. First, participants in middle adulthood slept less than younger and older participants, both based on self-reported as well as accelerometry-based information. Second, age-related differences in sleep efficiency were also related to the health status. Thus, the health status might be a highly relevant factor for explaining age differences in sleep quality.

## Discussion

The current study shows that accelerometry of body movements and posture during the night offers feasible, unobtrusive, reliable, and valid measurements of people’s nightly behavior in their natural environment. We first address unobtrusiveness and reliability and then discuss findings on convergent and external validity.

The self-reported sleep quality during the study night was high on average, and most participants felt undisturbed by the acceleration sensors. Only very few participants (5%) reported that they felt somewhat restricted by the equipment. Despite this, participants who felt restricted did not differ significantly from other participants in terms of sleep duration or self-reported sleep quality. It is important to note that, against participants’ subjective reports, acceleration-sensor information showed that they in fact lay for about a third of the night on their chest where sensors were attached.

The measurement of people’s posture and activity with sensors attached to the sternum and the thigh provided highly reliable measurements of whole body movements and thus posture classification. The newly developed algorithm that classified people’s posture based on the rotation of their main body axis additionally ensured the highly reliable classification of 99.7% correct body postures and changes of body posture.

Convergent validity between self-reported sleep duration and duration derived from posture classification and activity measurements implies that observing sleeping behavior with this posture-based approach is feasible and valid. The self-reported sleep duration did not significantly differ from sleep duration based on accelerometry. This is in line with other studies showing that sleep duration can be accurately determined by accelerometry [4], [18]. Importantly, the convergence between self-reported and posture-based sleep duration did not vary with participants’ age. This suggests that we were equally well able to measure sleep-related behavior from physical activity for people differing in age who also had a varied sleep quality. Previous reviews explicitly posed the question of whether sleep-focused accelerometry is equally well suited for heterogeneous samples. Associations with usual sleep duration were expectedly smaller because these ratings represent subjective averages of sleep duration over a longer period of time. These subjective averages typically differ from the sleep duration on certain nights and can also be biased by recall ability and/or general opinions and knowledge on the topic [28].

The results also showed some convergent validity between self-reported sleep quality and quality derived from nighttime accelerometry recordings. This is especially noteworthy because accelerometry-based indices possess no shared method variance with self-reported sleep quality, a factor that often enhances convergent validity. Since the correlation coefficients are small to moderate in size, further replication is necessary. Interestingly, activity-based indicators of sleep quality were more strongly related to general sleep quality, whereas judging the last night’s sleep as sufficient seemed to be based more on the time in bed, but less so on sleep-quality indicators. So far, only *wake after sleep onset* as objective measures of sleep quality has been related to subjective ratings of sleep quality in older people’s natural environment [29], [30]. Here, we present first evidence that posture-related indicators are linked to people’s subjective sleep quality, whereas previous laboratory studies linked longer sleep latency, more waking up, and lower sleep efficiency to sleep disorders [31], [32]. Thus, posture-related indicators might be a profitable route for future work on sleep quality within the normal population.

Both sleep duration and quality derived from assessments of posture and activity showed consistent external validity by replicating findings on sleeping behavior, for instance, the number of posture changes as well as on age differences in sleeping behavior. As in previous large-scale studies [7], [13] and comparable to the results from the representative German SOEP study for 2008, we also obtained a reported average sleep duration of about 7 hrs on week nights and observed that young and older adults both report and show longer sleep durations compared to middle-aged adults. Similarly, measurements of sleeping behavior with the accelerometry approach replicated known age differences in the quality of sleep behavior: Older people were less active during sleep, showed less posture changes, and arose less at the beginning of the night [16], [17]. Furthermore, we showed that age-related differences in sleep efficiency are related to age-related increases in illnesses. This concurs with a recent review of illnesses, and not age per se, being related to decreased sleeping quality [19].

### Strengths and Limitations

The strengths of the study lie in its assessment of sleep-related behavior of a sample larger and more heterogeneous than in average laboratory studies [9], in applying a newly developed posture-based approach to detect sleep-related behavior, and in the assessment within participants’ natural environment. The unobtrusive measurement likely increased participants’ acceptance and ensured ecologically valid assessments in people’s daily lives. Although wrist actigraphy needs only a watch-like device, our approach uses a similarly parsimonious instrumentation compared to polysomnography, especially when used with wireless acceleration sensors. In comparison to a polysomnographic approach, our equipment can be carried before and after nighttime, so that ambulatory assessment over longer periods is possible, for example, to assess daytime napping. Compared to studies using wrist actigraphy, we extended the scope of indicators usually derived from actigraphy and were able to reliably classify body postures using the newly developed approach. With this approach, we achieved a similar agreement between self-reported and activity-based sleep duration and quality as in recent studies using wrist actigraphy [29], [30], [33].

Nonetheless, there are a few limitations that have to be kept in mind. We did not compare the posture-based accelerometry to polysomnographic assessments in the laboratory because polysomnography has been questioned regarding the ecological validity [4], [5]. Future studies comparing accelerometry with ambulatory polysomnography need to keep in mind that the extensive polysomnographic instrumentation can alter the measured sleeping behavior even when applied in people’s natural environment. In contrast to current polysomnography, our approach did not distinguish specific sleeping phases. Long phases of inactivity in the supine posture during the nighttime strongly suggest that the observed person is asleep, but it is still possible that the person is reading, watching TV, or thinking. Future work might develop accelerometry-based indicators that distinguish between sleeping phases, similar to the approach using measurements of brain activity [34], [35]. Furthermore, as with wrist actigraphy, it is unlikely to distinguish whether the person is lying in bed or somewhere else, for instance, on a couch. This might, however, only be important for some research questions, but negligible for many others. Also, we only included participants without sleeping disorders, while clinical studies, for example, on sleep apnea, often focus on sleeping postures [36], [37]. For such studies, the classification approach presented here might, in fact, be even more important for monitoring sleeping postures in larger samples. For the comparison with subjective sleep quality, we used two questions regarding the study night and one global rating, which is very similar to the sleep-quality subscale from the established Pittsburgh Sleep Quality Index (PSQI) [29], [30], [38]. Future studies could assess the other subscales of the PSQI as well to achieve a more comprehensive measure of subjective sleep quality. Last, data from 12 participants were excluded due to technical problems that could not be resolved because the participants slept at home unattended. However, technical problems can occur in a similar vein for other ambulatory assessment methods, such as wrist actigraphy or polysomnography.

### Conclusions

Our findings show that monitoring sleep with ambulatory accelerometry yields results that are similar to subjective reports of sleep duration and quality in the participants’ natural environment for an age-heterogeneous sample. High subjective and objective sleep quality on the study night, as well as consistently observed validity, suggest that assessing sleep-related behavior with this newly developed posture-based approach is feasible. The low obtrusiveness and relatively low cost of the equipment compared to polysomnographic devices make it possible for researchers to assess sleep-related behavior in larger samples relevant for epidemiological studies. Today, wireless 3-D sensors, which start at a few hundred dollars, can be connected to smartphones, which are available for less than $500. Thus, although this equipment is more expensive than actigraphy watches, it offers more information regarding the posture and movement of the body. In addition to objective indicators of sleep duration, ambulatory accelerometry with sensors attached to the sternum and the thigh allow for a highly reliable classification of sleeping postures and computation of indicators for sleep-quality based on posture changes. The replication of age differences in sleep duration and quality so far observed in US or UK samples suggests that the current results could be generalizable to non-German populations. Altogether, this offers new possibilities for studying sleep postures and general sleep quality, which are proposed to be highly relevant for better understanding sleep-related mortality risks [3]. Therefore, posture-based ambulatory accelerometry indeed meets the initially stated aims of assessing sleep outside of laboratories to enhance ecological validity and reach larger, more representative samples.

## References

[1] Cappuccio FP, D’Elia L, Strazzullo P, Miller MA (2010) Sleep duration and all-cause mortality: A systematic review and meta-analysis of prospective studies. Sleep 33: 585–592. 20469800 10.1093/sleep/33.5.585 PMC2864873

[2] Gallicchio L, Kalesan B (2009) Sleep duration and mortality: A systematic review and meta-analysis. Journal of Sleep Research 18: 148–158. 19645960 10.1111/j.1365-2869.2008.00732.x

[3] Grandner MA, Hale L, Moore M, Patel NP (2010) Mortality associated with short sleep duration: The evidence, the possible mechanisms, and the future. Sleep Medicine Reviews 14: 191–203. 19932976 10.1016/j.smrv.2009.07.006 PMC2856739

[4] Ancoli-Israel S, Cole R, Alessi C, Chambers M, Moorcroft W, et al (2003) The role of actigraphy in the study of sleep and circadian rhythms. Sleep 26: 342–392. 12749557 10.1093/sleep/26.3.342

[5] Pollak CP, Tryon WW, Nagaraja H, Dzwonczyk R (2001) How accurately does wrist actigraphy identify the states of sleep and wakefulness? Sleep 24: 957–965. 11766166 10.1093/sleep/24.8.957

[6] Sadeh A (2011) The role and validity of actigraphy in sleep medicine: An update. Sleep Medicine Reviews 15: 259–267. 21237680 10.1016/j.smrv.2010.10.001

[7] Groeger JA, Zijlstra FRH, Dijk DJ (2004) Sleep quantity, sleep difficulties and their perceived consequences in a representative sample of some 2000 British adults. Journal of Sleep Research 13: 359–371. 15560771 10.1111/j.1365-2869.2004.00418.x

[8] Roberts RE, Shema SJ, Kaplan GA, Strawbridge WJ (2000) Sleep complaints and depression in an aging cohort: A prospective perspective. American Journal of Psychiatry 157: 81–88. 10618017 10.1176/ajp.157.1.81

[9] Ohayon MM, Carskadon MA, Guilleminault C, Vitiello MV (2004) Meta-analysis of quantitative sleep parameters from childhood to old age in healthy individuals: Developing normative sleep values across the human lifespan. Sleep 27: 1255–1273. 15586779 10.1093/sleep/27.7.1255

[10] Littner M, Kushida CA, Anderson WM, Bailey D, Berry RB, et al (2003) Practice parameters for the role of actigraphy in the study of sleep and circadian rhythms: An update for 2002. Sleep 26: 337–341. 12749556 10.1093/sleep/26.3.337

[11] Jean-Louis G, Von Gizycki H, Zizi F, Hauri P, Spielman A, et al (1997) The actigraph data analysis software: II. A novel approach to scoring and interpreting sleep–wake activity. Perceptual and Motor Skills 85: 219–226. 9293580 10.2466/pms.1997.85.1.219

[12] Jean-Louis G, Von Gizycki H, Zizi F, Hauri P, Spielman A, et al (1997) The actigraph data analysis software: I. A novel approach to scoring and interpreting sleep–wake activity. Perceptual and Motor Skills 85: 219–226. 9293580 10.2466/pms.1997.85.1.219

[13] Krueger PM, Friedman EM (2009) Sleep duration in the United States: A cross-sectional population-based study. American Journal of Epidemiology 169: 1052–1063. 19299406 10.1093/aje/kwp023 PMC2727237

[14] Giganti F, Ficca G, Gori S, Salzarulo P (2008) Body movements during night sleep and their relationship with sleep stages are further modified in very old subjects. Brain Research Bulletin 75: 66–69. 18158097 10.1016/j.brainresbull.2007.07.022

[15] De Koninck J, Lorrain D, Gagnon P (1992) Sleep positions and position shifts in five age groups: An ontogenetic picture. Sleep 15: 143–149. 1579788 10.1093/sleep/15.2.143

[16] Lorrain D, De Koninck J, Dionne H, Goupil G (1986) Sleep positions and postural shifts in elderly persons. Perceptual and Motor Skills 63: 352–354. 3774439 10.2466/pms.1986.63.2.352

[17] Gori S, Ficca G, Giganti F, Di Nasso I, Murri L, et al (2004) Body movements during night sleep in healthy elderly subjects and their relationships with sleep stages. Brain Research Bulletin 63: 393–397. 15245766 10.1016/j.brainresbull.2003.12.012

[18] Kline CE, Zielinski MR, Devlin TM, Kripke DF, Bogan RK, et al (2010) Self-reported long sleep in older adults is closely related to objective time in bed. Sleep and Biological Rhythms 8: 42–51. 25210491 10.1111/j.1479-8425.2009.00422.x PMC4157821

[19] Vitiello MV (2009) Recent advances in understanding sleep and sleep disturbances in older adults: Growing older does not mean sleeping poorly. Current Directions in Psychological Science 18: 316–320.

[20] Mehra R, Stone KL, Blackwell T, Ancoli Israel S, Dam TTL, et al (2007) Prevalence and correlates of sleep-disordered breathing in older men: Osteoporotic fractures in Men Sleep Study. Journal of the American Geriatrics Society 55: 1356–1364. 17767677 10.1111/j.1532-5415.2007.01290.x PMC2780325

[21] Mathie MJ, Celler BG, Lovell NH, Coster ACF (2004) Classification of basic daily movements using a triaxial accelerometer. Medical & Biological Engineering & Computing 42: 679–687. 15503970 10.1007/BF02347551

[22] Quinlan JR (1986) Induction of decision trees. Machine Learning 1: 81–106.

[23] Mathie MJ, Coster ACF, Lovell NH, Celler BG (2004) Accelerometry: Providing an integrated, practical method for long-term, ambulatory monitoring of human movement. Physiological Measurement 25: 1–20. 15132305 10.1088/0967-3334/25/2/r01

[24] Wagner GG, Frick JR, Schupp J (2007) The German Socio-Economic Panel Study (SOEP)–Scope, evolution and enhancements. Schmollers Jahrbuch: Journal of Applied Social Science 127: 139–169.

[25] Gordon SJ, Grimmer KA, Trott P (2004) Self reported versus recorded sleep position: An observational study. The Internet Journal of Allied Health Sciences and Practice 2: 1–10.

[26] Lorrain D, De Koninck J (1998) Sleep position and sleep stages: Evidence of their independence. Sleep 21: 335–340. 9646377 10.1093/sleep/21.4.335

[27] Taylor MF (1993) The British Household Panel Study. ESRC Data Archive Bulletin 53: 7–10. 12287398

[28] Furnham A, Henderson M (1982) The good, the bad and the mad: Response bias in self-report measures. Personality and Individual Differences 3: 311–320.

[29] Beaudreau SA, Spira AP, Stewart A, Kezirian EJ, Lui L-Y, et al (2012) Validation of the Pittsburgh Sleep Quality Index and the Epworth Sleepiness Scale in older black and white women. Sleep Medicine 13: 36–42. 22033120 10.1016/j.sleep.2011.04.005 PMC3586742

[30] Spira AP, Beaudreau SA, Stone KL, Kezirian EJ, Lui LY, et al (2012) Reliability and validity of the Pittsburgh Sleep Quality Index and the Epworth Sleepiness Scale in older men. Journals of Gerontology Series A: Biological Sciences & Medical Sciences 67A: 433–439. 10.1093/gerona/glr172 PMC3309871 21934125

[31] De Koninck J, Gagnon P, Lallier S (1983) Sleep positions in the young adult and their relationship with the subjective quality of sleep. Sleep 6: 52–59. 6844798 10.1093/sleep/6.1.52

[32] Sahlin C, Franklin KA, Stenlund H, Lindberg E (2009) Sleep in women: Normal values for sleep stages and position and the effect of age, obesity, sleep apnea, smoking, alcohol and hypertension. Sleep Medicine 10: 1025–1030. 19345643 10.1016/j.sleep.2008.12.008

[33] Lauderdale DS, Knutson KL, Yan LL, Liu K, Rathouz PJ (2008) Self-reported and measured sleep duration: How similar are they? Epidemiology 19 838–845: 810.1097/EDE.1090b1013e318187a318187b318180. 10.1097/EDE.0b013e318187a7b0 PMC2785092 18854708

[34] Feinberg I, Keresko RL, Heller N (1967) EEG sleep patterns as a function of normal and pathological aging in man. Journal of Psychiatric Research 5: 107–144. 6056816 10.1016/0022-3956(67)90027-1

[35] Kahn E, Fisher C (1970) Some correlates of rapid eye movement sleep in the normal aged male. Journal of Nervous and Mental Disease 148: 495–505. 10.1097/00005053-196905000-00003 4305828

[36] Richard W, Kox D, Den Herder C, Laman M, Van Tinteren H, et al (2006) The role of sleep position in obstructive sleep apnea syndrome. European Archives of Oto-Rhino-Laryngology 263: 946–950. 16802139 10.1007/s00405-006-0090-2

[37] Walsh JH, Leigh MS, Paduch A, Maddison KJ, Armstrong JJ, et al (2008) Effect of body posture on pharyngeal shape and size in adults with and without obstructive sleep apnea. Sleep 31: 1543–1549. 19014074 10.1093/sleep/31.11.1543 PMC2579983

[38] Buysse DJ, Reynolds CF, Monk TH, Berman SR, Kupfer DJ (1989) The Pittsburgh Sleep Quality Index: A new instrument for psychiatric practice and research. Psychiatry Research 28: 193–213. 2748771 10.1016/0165-1781(89)90047-4

